# Enhancing Tumor Photodynamic Therapy via Molecular Engineering and Functional Modification of Photosensitizers

**DOI:** 10.3390/molecules31030560

**Published:** 2026-02-05

**Authors:** Wei Zheng, Linlin Tao, Xiaofeng Xia, Tianlin Wang, Feiyi Wang

**Affiliations:** 1 College of Chemistry and Chemical Engineering, Anshun University, Anshun 561000, China; 18386422711@163.com; 2 Hubei Key Laboratory of Drug Synthesis and Optimization, Jingchu University of Technology, Jingmen 448000, China; tlljclg@163.com (L.T.); 202412012@jcut.edu.cn (X.X.); 3 Hubei Three Gorges Laboratory, Yichang 443007, China; 4 Hubei Key Laboratory for Precision Synthesis of Small Molecule Pharmaceuticals & Ministry of Education Key Laboratory for the Synthesis and Application of Organic Functional Molecules, Hubei University, Wuhan 430062, China

**Keywords:** photosensitizers, photodynamic therapy, aggregation-induced emission, functionalizing, targeting groups

## Abstract

Photosensitizers are susceptible to interference from the biological internal environment, which largely restricts the clinical application of photodynamic therapy. For instance, most existing photosensitizers tend to aggregate in the biological environment, resulting in a decrease in reactive oxygen species yield; their therapeutic efficacy is unsatisfactory in hypoxic tumor environments; they are difficult to accumulate effectively in tumor sites and cannot accurately distinguish between tumors and healthy tissues. To address these issues, this review systematically elaborates on a series of optimization strategies, including improving the intersystem crossing efficiency of photosensitizers through molecular engineering, endowing them with aggregation-induced emission properties, developing type I photosensitizers, and functionalizing photosensitizers by modifying biological proteins, targeting groups, or combining with nanoengineering, aiming to enhance the efficiency of photodynamic therapy. By summarizing the latest research breakthroughs, innovative methods, and emerging applications in this field, the review provides practical solutions and broad application prospects for photodynamic therapy, which is expected to promote the clinical translation and application of photosensitizers.

## 1. Introduction

**Figure 1:**
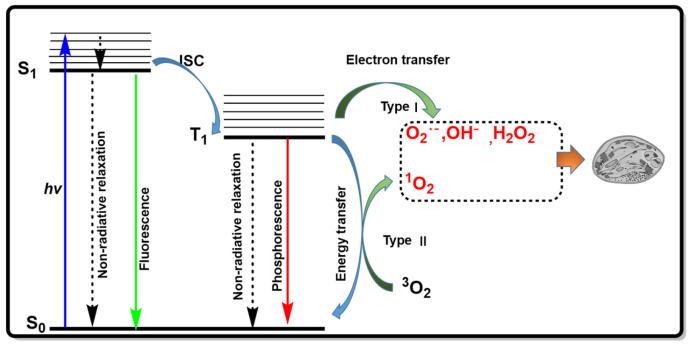
Jablonski diagram illustrating type I and type II PDT mechanisms.

Cancer is characterized by its high incidence rate, lack of early-stage symptoms, low cure rates, and tendencies toward invasion and metastasis, making it one of the most lethal diseases globally and a persistent threat to human health [1,2,3]. Treating cancer is a daunting challenge. While refining conventional therapies (such as chemotherapy, radiotherapy, and surgery) is important, there is an urgent need to develop novel treatment modalities that enhance efficacy while minimizing side effects [4,5,6]. Photodynamic therapy (PDT) has emerged as a highly promising cancer treatment due to its precision, minimal invasiveness, and low systemic toxicity, and broad spectrum of therapeutic applications, making it a critical area of research in pharmaceutical science in recent decades [7,8,9]. During PDT, Photosensitizers (PSs) generate reactive oxygen species (ROS) (including singlet oxygen (^1^O_2_), superoxide radicals (O_2_^•−^), hydroxyl radicals (•OH), and hydrogen peroxide (H_2_O_2_)) only under specific wavelengths of light and oxygen conditions. These ROS are cytotoxic and can induce cell apoptosis and death, thereby achieving tumor suppression [10,11]. Upon irradiation, the PS is excited from the ground state (S_0_) to the excited singlet state (*S*_1_). This excited state has a short lifetime and rapidly relaxes back to S_0_ by emitting fluorescence. For ROS generation, the *S*_1_ first undergoes intersystem crossing (ISC) to the triplet state (*T*_1_). In type I reactions, •OH and O_2_^•−^ are produced via photo-induced electron transfer between the PS’s *T*_1_ and substrates. In type II reactions, ^3^O_2_ is converted into highly reactive ^1^O_2_ through energy transfer (Figure 1) [12]. Since •OH and O_2_^•−^ exhibit higher cytotoxicity than ^1^O_2_ and their generation is less oxygen-dependent, type I mechanisms are particularly effective for PDT in hypoxic tumors [13,14].

Despite its advantages, PDT still faces several critical challenges: (1) Conventional organic PSs—such as porphyrins [15,16], boron-dipyrromethene (BODIPY) [17,18], and phthalocyanine derivatives [19,20] are large conjugated aromatic compounds that exhibit strong ROS generation in solution. However, they tend to undergo π-π stacking aggregation in aqueous solutions, exerting the opposite effect. This well-known phenomenon is referred to as the aggregation-caused quenching (ACQ) effect [21,22]. (2) The PDT process requires significant oxygen consumption, yet deep tumors are often hypoxic with extremely low oxygen concentrations. This severely limits ROS generation and diminishes therapeutic efficacy [14,23,24]. (3) Most of the existing PSs exhibit weak tissue penetration ability and lack active tumor-targeting capability, which hinders their selective accumulation in cancer cells and impairs the therapeutic efficacy [25,26]. Given these limitations, improving the therapeutic efficacy of PSs in PDT remains critically important.

To address these challenges, researchers are actively exploring effective solutions. Although combination strategies and auxiliary approaches are of great value, optimizing the performance and functions of PSs remains one of the most critical pathways [27,28,29]. The core strategies adopted in this study are summarized as follows: (1) Enhancing ISC efficiency via molecular engineering to improve the quantum yield of ROS: The ISC efficiency of molecules can be improved by introducing heavy atoms or carbonyl groups at specific molecular sites [30,31]. (2) Utilizing aggregation-induced emission (AIE) PSs: Unlike traditional ACQ agents, AIE PSs exhibit minimal emission in monomeric states but demonstrate strong fluorescence and enhanced ROS production in aggregated states [32,33]. (3) Designing type I PSs: This category of PSs reduces oxygen dependence and can efficiently generate ROS even in the hypoxic tumor microenvironment [8,34,35,36]. (4) Modifying tumor-targeting moieties: Targeting groups enhances the specificity and accumulation of PS in tumors to improve PDT efficacy [37,38,39,40,41,42,43,44]. Finally, this paper outlines the prospects for addressing current challenges and future development directions, which include constructing more stable PS carriers, PSs with low oxygen dependence, combination therapies, and multifunctional nanoplatforms [45,46,47,48,49].

## 2. The Increase in ROS Production by Improving the ISC Rate

In conventional PSs, porphyrins, BODIPY, and phthalocyanine derivatives have been widely employed in PDT due to their high molar absorption coefficients, fluorescence emission, and ROS generation capabilities. However, the challenge associated with precisely monitoring and regulating ROS production has impeded its prospective clinical translation [50,51]. The ISC efficiency of PSs serves as a critical determinant of ROS generation yield. When PSs are in the *T*_1_, they transfer energy and electrons to surrounding substrates and ^3^O_2_, thereby producing ROS. This makes the *T*_1_ fundamentally important for PS performance. Since the *T*_1_ is populated through non-radiative ISC transitions from the *S*_1_, enhancing the ISC rate can significantly improve ROS production efficiency [31,52]. The ISC rate constant (*k_ISC_*) is defined by Equation (1):

(1) $$k_{ISC}\;\propto \frac{{<T_{1}|H_{SO}|S_{1}>}^{2}}{(\Delta E_{ST}{)}^{2}}$$

where Δ*E_ST_* is the energy gap between *S*_1_ and *T*_1_, and ⟨*T*_1_|*H_SO_*|*S*_1_⟩ represents the spin–orbit coupling (SOC) matrix element [53]. Consequently, reducing Δ*E_ST_* or strengthening SOC can accelerate ISC rates, thereby promoting *S*_1_ → *T*_1_ transitions and ultimately enhancing ROS generation.

### 2.1. Enhancing SOC for Improved PDT Efficacy

The introduction of heavy atoms into PSs significantly enhances SOC, thereby accelerating ISC rates and substantially improving ROS generation. Numerous studies have demonstrated that incorporating Br or I into the chromophores of porphyrins, BODIPY, phthalocyanines, and their derivatives effectively increases ROS quantum yields [54,55,56]. This modification not only maintains the planarity of the conjugated π-system but also enhances the ISC process and ^1^O_2_ generation through SOC interactions. In addition, the heavy atom effect can induce a redshift in the absorption spectrum of PSs; since light scattering is reduced at longer wavelengths and biological tissues exhibit lower absorption of long-wavelength light, this redshift facilitates deeper tissue penetration. In addition, although the heavy atom effect quenches fluorescence, it can accelerate the ISC process to the *T*_1_ state, which is crucial for improving the ROS production efficiency.

**Figure 2:**
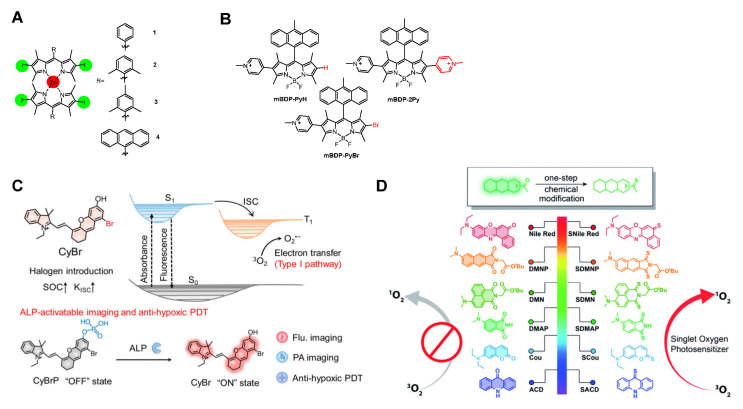
(**A**) Chemical structures of iodinated dipyrrinato Zn(II) complexes 1–4. (**B**) Chem ical structures of BODIPY derivatives mBDP-PyH, mBDP-2Py, and mBDP-2PyBr. (**C**) The “off” state of CyBrP is activated to the “ON” state by ALP, and then applied for PDT therapy. Reprinted with permission from Ref. [59]. Copyright 2023 American Chemical Society. (**D**) Thiocarbonyl substitution at carbonyl groups of fluorophores enhances ROS generation capability.

Gasser et al. [57] reported iodinated dipyrrinato Zn(II) complexes (1–4) exhibiting long *T*_1_ lifetimes (τ_T_ = 207–559 ns) and efficient ^1^O_2_ generation. The introduction of iodine significantly enhanced SOC, suppressing radiative decay pathways while promoting ISC efficiency. Notably, compound 3 demonstrated enhanced photocytotoxicity under light irradiation (Figure 2A). Liu et al. [58] strategically introduced bromine into BODIPY derivatives to modulate the PS’s *T*_1_ lifetime and ISC efficiency. Building upon a previously reported near-infrared (NIR)-emissive BODIPY skeleton, they developed three novel PSs by incorporating bromine and pyridinium salt moieties. The bromine enhanced SOC via the heavy-atom effect, accelerating ISC and boosting ROS generation, while the pyridinium salt further amplified this process. The resulting mBDP-PyBr exhibited strong NIR emission, enabled multimodal ROS generation, and achieved remarkable PDT efficacy even under hypoxic conditions (Figure 2B). Ma et al. [59] engineered a type I PS (CyBr) by substituting bromine into a hemicyanine (Cy) scaffold. Conventional Cy exhibits low ROS yields, whereas bromine incorporation elevated the SOC constant, enhancing ISC rates to boost type I ROS generation (Figure 2C). While heavy-atom incorporation enhances ISC processes, it often increases dark toxicity, necessitating the development of heavy atom-free PSs [60].

Over recent decades, sulfur-based or thio-substituted moieties have gained attention due to their favorable phototherapeutic properties, leading to extensive applications in anticancer agents [61,62]. Thiocarbonyl groups, for instance, effectively populate *T*_1_ by strengthening SOC, thereby boosting ISC efficiency.

**Figure 3:**
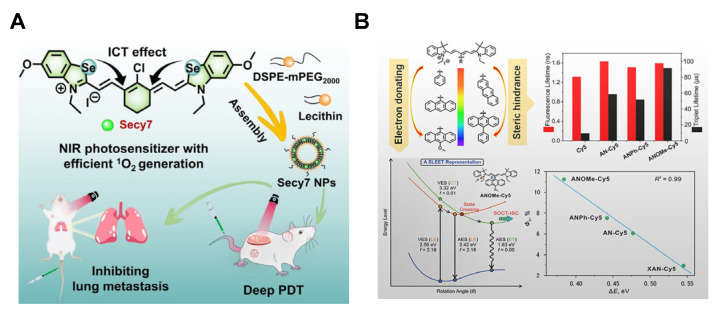
(**A**) PDT mechanism of selenium-incorporated heptamethine cyanine PSs. Reprinted with permission from Ref. [64]. Copyright 2024 Wiley-VCH. (**B**) Design of heavy-atom-free PSs by introducing sterically bulky and electron-rich moieties at the meso position of the Cy5 skeleton with improved ISC rate and prolonged excited-state lifetime. Reprinted with permission from Ref. [70]. Copyright 2021 American Chemical Society.

Xiao et al. [63] developed a series of heavy atom-free PSs by replacing oxygen with sulfur in conventional fluorophores for visible-to-NIR PDT. These PSs exhibited high phototoxicity against HeLa cells with negligible dark toxicity (Figure 2D). Peng et al. [64] reported NIR PSs based on sulfur- and selenium-incorporated heptamethine cyanine dyes with high ^1^O_2_ quantum yields. The heptamethine cyanine scaffold provided strong NIR absorption and a high molar extinction coefficient. Introducing sulfur and selenium enhanced SOC via the heavy-atom effect, while strong intramolecular charge transfer (ICT) between selenium and the polymethine chain narrowed the energy gap (Δ*E_ST_* = 0.51 eV). These synergistic effects increased the *T*_1_ state population up to 61%, facilitating efficient energy transfer to molecular oxygen and dramatically enhancing ^1^O_2_ generation (Figure 3A). Additionally, metal complexes (e.g., Ru(II), Ir(III), Pt(II), Au(I), and Os(II)) can be integrated into PSs to enhance SOC and ISC efficiency [65,66,67].

### 2.2. Reducing the ΔE_ST_ for Improved PDT Efficacy

The enhancement of SOC is typically achieved by incorporating heavy atoms (e.g., Se, Br, or I) into PSs. However, these heavy-atom-containing PSs often exhibit inherent cytotoxicity and non-degradability, limiting their biological applications. To address these limitations, minimizing the Δ*E_ST_* has emerged as an effective alternative strategy to enhance ISC efficiency. This can be realized by designing conjugated molecular systems through donor–acceptor (D-A) combinations, which modulate electronic structures to reduce Δ*E_ST_* [68,69].

**Figure 4:**
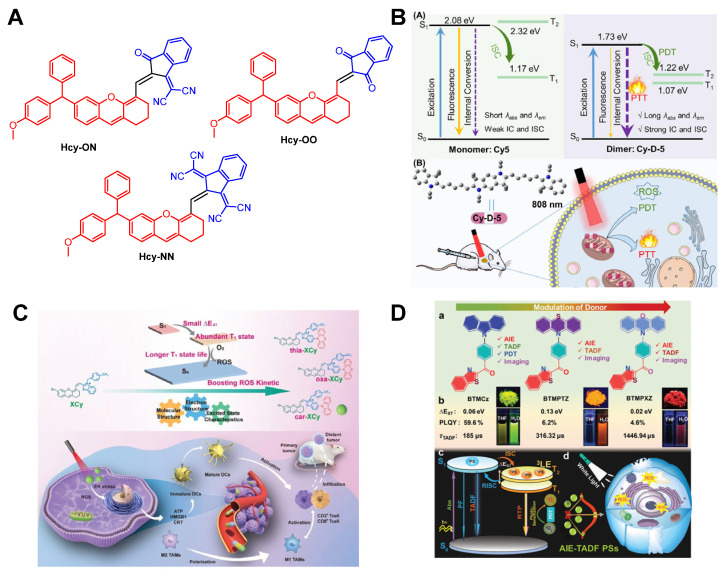
(**A**) Chemical structure diagrams of Hcy-ON, Hcy-OO and Hcy-NN. (**B**) The pentamethylene cyanine dimer decreases the Δ*E_ST_* in Cy-D-5 and thus enhances the efficiency of PDT. (**C**) Schematic diagram of high-efficiency ROS-generating PDT for XCys. Reprinted with permission from Ref. [78]. Copyright 2024 Wiley-VCH. (**D**) Optical properties and applications of BTMCz, BTMPTZ and BTMPXZ in PDT Reprinted with permission from Ref. [79]. Copyright 2025 Wiley-VCH.

Peng et al. [70] introduced sterically bulky and electron-rich moieties at the meso-position of a cyanine-based skeleton. This design reduced the spatial overlap between the highest occupied molecular orbital (HOMO) and the lowest unoccupied molecular orbital (LUMO), thereby lowering Δ*E_ST_*. The reduced energy gap enhanced ISC efficiency and prolonged the triplet-state lifetime, significantly improving ^1^O_2_ generation (Figure 3B). Li et al. [71] employed an acceptor engineering strategy to reduce the Δ*E_ST_* of the Hcy-ON by incorporating diphenylamine donors and varying numbers of cyano groups into a xanthene structure. This molecular design enabled precise tuning of both excitation wavelengths and Δ*E_ST_* values. The optimized Hcy-ON exhibited the smallest Δ*E_ST_* (0.678 eV) between the *S*_1_ and *T*_1_, which significantly enhanced ISC efficiency. Under 760 nm laser irradiation, Hcy-ON demonstrated high ROS generation and robust photothermal conversion, enabling synergistic photodynamic/photothermal therapy in hypoxic tumor (Figure 4A). Chen et al. [72] reported that dimeric pentamethine cyanine (Cy-D-5) achieves an ultra-low Δ*E_ST_* (0.51 eV) compared to conventional cyanine dyes. Cy-D-5 exhibited a non-radiative transition rate 12.6-fold higher than indocyanine green (ICG), along with enhanced ^1^O_2_ generation, photothermal conversion efficiency (η = 64.4%), and photostability (Figure 4B). Although these strategies can effectively reduce the Δ*E_ST_*, such methods lack universality and cannot be applied on a large scale.

In recent years, metal-free organic small molecules with ultra-small Δ*E_ST_* (0.01–0.05 eV), termed thermally activated delayed fluorescence (TADF) emitters, have been developed for organic light-emitting diodes (OLEDs). These materials leverage reverse intersystem crossing (RISC) enabled by minimized Δ*E_ST_*, achieving near-unity internal quantum efficiency without precious metals [73,74]. By modulating electron-donating or withdrawing groups in TADF molecules, HOMO and LUMO energy levels can be precisely adjusted to tailor Δ*E_ST_* for optimized RISC dynamics [75,76,77]. Zhong et al. [78] designed a TADF-based PS (car-XCy) with optimized excited-state characteristics, achieving a 100-fold increase in triplet-state lifetime and a 225% improvement in ROS yield compared to the parent XCy (Figure 4C). Iyer et al. [79] demonstrated PSs integrating TADF and AIE properties, enabling precise control of excited-state dynamics via second-order spin–orbit perturbation mechanisms. Among these, the BTMCz derivative balanced an ultra-small Δ*E_ST_* (≤0.05 eV), which is conducive to improving the generation efficiency of ^1^O_2_ (Figure 4D). Although this method holds promising application prospects, its core limitation lies in the intense competition between the radiative transitions responsible for luminescence and the non-radiative transitions involved in ROS generation, which remains a key challenge that researchers need to overcome.

## 3. Engineering AIE PSs to Enhance PDT

Most existing PSs are confronted with the critical issue of insufficient ROS generation in aqueous media. A key challenge arises from their ACQ behavior, where π-π stacking and intermolecular interactions in aggregated states suppress fluorescence and ROS production, severely restricting PDT efficacy [80]. In contrast, AIE materials, pioneered by Tang et al., exhibit diametrically opposite behavior: AIE-active PSs display negligible emission in molecularly dissolved states but achieve bright fluorescence and enhanced ROS generation upon aggregation. This phenomenon stems from restricted intramolecular motion (RIM) and suppressed non-radiative decay pathways in aggregated or solid-state environments [14,81,82,83].

**Figure 5:**
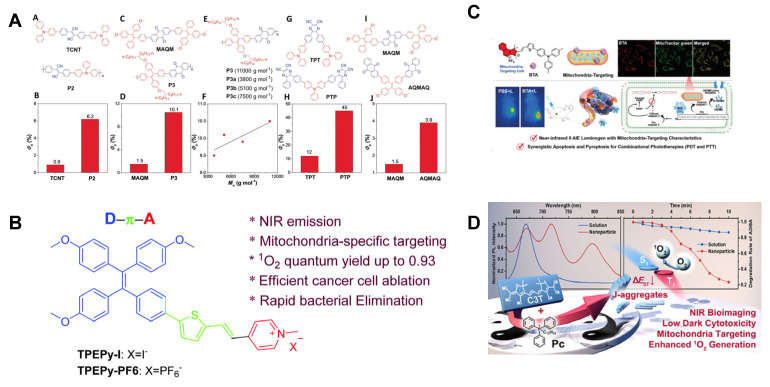
(**A**) Chemical structure of various PSs with D-A structures and their ROS yield. Reprinted with permission from Ref. [84]. Copyright 2018 Wiley-VCH. (**B**) Molecular structures and photophysical properties of two cationic PSs exhibiting AIE characteristics. Reprinted with permission from Ref. [85]. Copyright 2020 Royal Society of Chemistry. (**C**) Schematic illustration of NIR-II AIE PS with mitochondrial targeting capability for breast cancer therapy. Reprinted with permission from Ref. [86]. Copyright 2024 Wiley-VCH. (**D**) Enhanced PDT efficacy through anionic cyanine-based *J*-type aggregation. Reprinted with permission from Ref. [87]. Copyright 2022 Wiley-VCH.

Traditional donor–acceptor (D-A)-type PSs exhibit tunable photosensitizing activity through modifications to electron-donating (D) or electron-withdrawing (A) groups. Tang et al. [84] demonstrated that polymerizing D-A-type AIE-active PSs or adjusting the D/A ratio significantly enhances photosensitizing efficiency. They designed multiple compounds and quantified ^1^O_2_ generation. For TCNT, polymerized forms achieved a ROS yield of 6.2%, a 5.8-fold increase compared to its monomeric counterpart (0.9%). Similarly, the polymerized version of MAQA showed a 5.7-fold enhancement in ROS yield over its monomer. Additionally, increasing the proportion of A units in D-A architectures improved efficiency: PTP (A-D-A structure) exhibited a ROS yield of 45%, 2.7 times higher than TPT (D-A-D structure, 12%). AQMAQ (A-D-A structure) achieved a ROS yield of 3.9%, outperforming MAQM (D-A-D structure, 1.5%). These results highlight that increasing the proportion of A units in D-A molecular architectures enhances photosensitizing efficiency (Figure 5A). Chen et al. [85] reported two cationic AIE PSs (TPEPy-I and TPEPy-PF6) with identical backbones but distinct counterions (I^−^ vs. PF_6_^−^). Both PSs exhibited a strong push–pull electronic effect, NIR emission, enhanced ^1^O_2_ generation capability, and mitochondria-targeting aggregation and fluorescence turn-on behavior. Under white light irradiation, these PSs effectively inhibited tumor cell proliferation and bacterial growth via ROS-mediated mechanisms (Figure 5B). AIE PSs not only effectively enhance the ROS generation efficiency but also facilitate the development of PSs with long-wavelength emission and enhanced fluorescence brightness.

PSs with long-wavelength emission are critical for the diagnosis and treatment of deep-seated tumors and visceral diseases, significantly enhancing therapeutic efficacy. This spectral optimization improves the precision of PDT by allowing deeper tissue penetration and spatially resolved activation. Tan et al. [86] reported a NIR-II AIE PS (BTA) with mitochondrial targeting properties for the treatment of breast tumors. By systematically modulating the electron-donating strength of the donor moiety in BTA, the team achieved intense NIR-II fluorescence (1000–1700 nm) with distinct AIE characteristics, enabling precise targeting of tumor cell mitochondria. Experimental results demonstrate that BTA efficiently generates both type I and type II ROS while exhibiting photothermal effects in vitro and in vivo (Figure 5C).

While most ionic PSs are cationic and exhibit excellent properties, some anionic PSs also demonstrate significant advantages, including NIR emission, high photostability, negatively charged surfaces, targeting ability, and significantly improved ^1^O_2_ production efficiency. Li et al. [87] developed an anionic cyanine-based *J*-aggregate PS with enhanced photodynamic efficacy through counterion engineering. By introducing a typical mitochondrial targeting agent—dodecyl (triphenyl) phosphonium cation (Pc)—they obtained C3T-Pc, which forms *J*-type aggregates in nanoparticles. This system exhibits NIR emission, high photostability, and mitochondrial targeting ability, while demonstrating enhanced ROS production that effectively inhibits tumor growth (Figure 5D). Although AIE photosensitizers can enhance the efficacy of photodynamic therapy (PDT), specific strategies are required for their application in hypoxic tumors.

## 4. Engineering Type I PSs to Enhance PDT

Most existing photosensitizers require sufficient oxygen to generate singlet oxygen, and thus exhibit poor therapeutic efficacy in hypoxic tumors, which has greatly hindered their clinical translation and application. However, type I PSs with the ability to generate free radical ROS have become a strong candidate for overcoming the inherent hypoxic characteristics of solid tumors [88,89,90,91].

**Figure 6:**
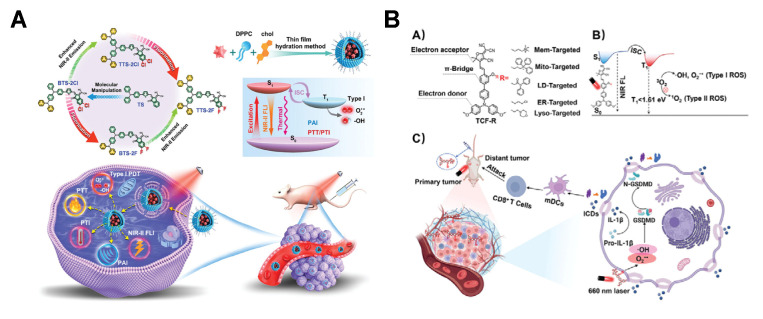
(**A**) Schematic diagram of NIR-II AIE liposome-enhanced type I PDT for breast cancer. Reprinted with permission from Ref. [93]. Copyright 2024 Wiley-VCH. (**B**) Organelle-targeting NIR-type I PSs for PDT applications. Reprinted with permission from Ref. [94]. Copyright 2024 Wiley-VCH.

Type I PSs can enhance ISC and facilitate electron transfer via molecular design involving the incorporation of strong electron-acceptor groups and heteroatoms (or heterocycles), which ultimately leads to an increased generation of type I ROS [24,92]. Tan et al. [93] reported that NIR-II AIE a liposome enhanced type I PDT and mild-temperature photothermal therapy (PTT) for breast cancer. The introduction of F atoms and DPA groups into PSs exhibits high twisted configurations, low-frequency vibrations, small Δ*E_ST_*, and strong D-A interactions. These features consequently inhibit the energy transfer process of PS and boost the capacity for generating ROS (Figure 6A). Gong et al. [94] reported a series of organelle-targeting NIR type I PSs through molecular engineering. By employing tricyanofuran (TCF) as an electron acceptor, phenol as a π-bridge, and triphenylamine as an electron donor, they constructed five AIE-active PSs targeting distinct subcellular organelles. Theoretical calculations revealed that these PSs exhibit low *T*_1_ energy levels (<1.61 eV), which minimize energy dissipation pathways and optimize electron transfer processes. This design facilitates type I ROS generation rather than oxygen-dependent type II ROS, significantly enhancing therapeutic efficacy against hypoxic solid tumors (Figure 6B).

**Figure 7:**
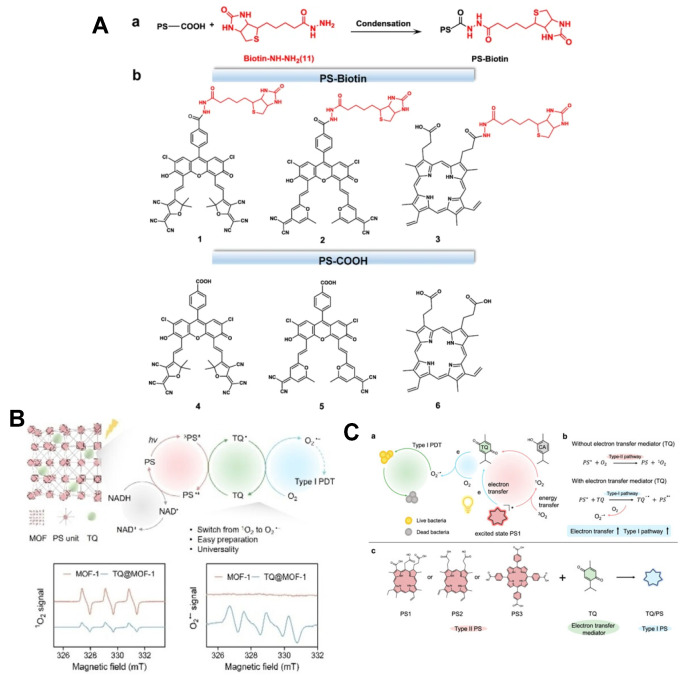
(**A**) Structural diagrams of three traditional photosensitizers and three biotin-modified PSs. (**B**) Design of porphyrin-based MOFs for TQ-mediated type II-to-type I PS conversion. Reprinted with permission from Ref. [96]. Copyright 2024 Wiley-VCH. (**C**) Schematic diagram of carvacrol-promoted of O_2_^•−^ generation by PSs.

Electron-deficient structural intermediates can effectively convert type II PSs into type I PSs, or increase the proportion of type I ROS [24]. Zheng et al. [95] reported a method for converting type II PSs into type I PSs. Three traditional PSs (two based on fluorescein and commercially available protoporphyrin) can all produce ^1^O_2_, which is a typical type II PSs. However, the PS enhances their ability to generate free radical ions by amidating biotin and can simultaneously produce a large amount of O_2_^•−^ (Figure 7A). Liu et al. [96] reported a series of porphyrin-based MOFs that were transformed from the TQ-mediated type II pathway to the type I pathway. TQ, as an electron transfer medium, can accept electrons generated by porphyrin MOFs in an excited state and transfer them to the vicinity of oxygen, thereby producing a large amount of O_2_^•−^. TQ@MOF-1 nanoparticles exhibit strong in vitro phototoxicity under low oxygen conditions (Figure 7B). Liu et al. [97] reported that the natural substrate carvacrol (CA) promotes the conversion of typical type II PSs to type I PSs, generating O_2_^•−^. The local ^1^O_2_ generated by PSs under light irradiation converts CA into TQ. TQ, as an efficient electron transfer mediator, promotes electron transfer through the type I pathway of PSs, thereby generating a large amount of superoxide radicals (Figure 7C). While type I PSs can effectively overcome the therapeutic bottleneck of hypoxic tumors, their efficient accumulation at tumor sites and the reduction in toxic side effects on healthy tissues remain critical challenges to be addressed urgently.

## 5. Advancing PDT Through Functionalized PSs

The human body contains abundant biomolecules such as polysaccharides, proteins, peptides, and antibodies. By coupling therapeutic agents with these biomolecules, not only can biocompatibility be improved, but enhanced targeting and specific selectivity can also be achieved, thereby reducing immune resistance and toxic side effects [98,99]. The core challenges of this strategy lie in achieving precise delivery, efficient accumulation, and safe controllability. Its design principles mainly include two aspects: first, modifying stimulus-responsive groups to enable specific activation of therapeutic agents at lesion sites; second, constructing carrier systems with specific functions to improve the performance and targeting capability of photosensitizers.

### 5.1. Active-Targeting PSs for Precision PDT

Most reported PSs enhance their targeting ability by utilizing functional groups that recognize specific biomarkers within the tumor microenvironment or organelles. This targeted approach improves PS accumulation at tumor sites while minimizing damage to healthy tissues, thereby enhancing treatment safety [100,101,102,103,104,105].

**Figure 8:**
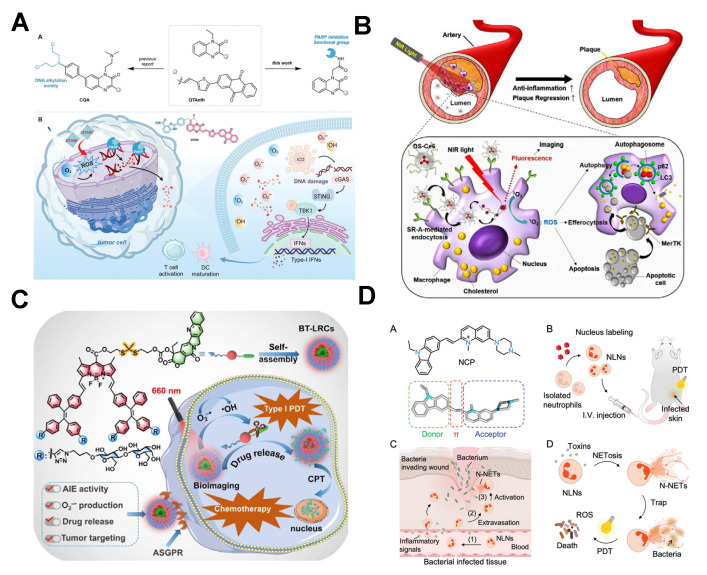
(**A**) Schematic diagram of PSs targeting and inhibiting poly (ADP-ribose) polymerase activity for PDT. Reprinted with permission from Ref. [108]. Copyright 2024 Wiley-VCH. (**B**) Schematic diagram of PSs targeting macrophages for atherosclerosis treatment. (**C**) Schematic diagram of tumor-targeted therapy using glycosylated PSs. Reprinted with permission from Ref. [110]. Copyright 2024 Wiley-VCH. (**D**) Schematic diagram of nuclear-targeting PSs for photodynamic treatment of multidrug-resistant bacterial infections. Reprinted with permission from Ref. [111]. Copyright 2024 Wiley-VCH.

Rationally designed photosensitizers can achieve specific organelle targeting, activate organelle-mediated cell death pathways, maximize the local oxidative damage effect, and significantly reduce systemic toxicity while boosting therapeutic efficacy [106,107]. Shi et al. [108] developed a QTABI that specifically targets and inhibits poly (ADP-ribose) polymerase (PARP). Under light irradiation, QTABI generates substantial ROS to induce DNA damage while blocking the DNA damage response through PARP inhibition, leading to tumor cell apoptosis/necrosis (Figure 8A). Kim et al. [109] designed a dextran sulfate-chlorin e6 conjugate (DS-Ce6) for macrophage targeting. DS-Ce6 is internalized via scavenger receptor A (SR-A)-mediated endocytosis into activated macrophages and foam cells. Light activation of DS-Ce6 reduced atherosclerotic plaque burden and inflammation in murine models (Figure 8B). Xing et al. [110] synthesized a lactosylated BT-LRC by covalently linking camptothecin (CPT) to a BODIPY-TPE derivative via a thioketal bond. BT-LRC self-assembles into nanoparticles (BT-LRCs) that target HepG2 cells through asialoglycoprotein receptor (ASGPR)-mediated endocytosis. Upon irradiation, BT-LRCs generate O_2_^•−^ to cleave the thioketal bond, releasing CPT for synergistic therapy (Figure 8C). Mao et al. [111] developed a nucleus-targeting NCP containing a quinolinium salt for DNA binding and an N-ethyl carbazole-conjugated system. The spatial separation of HOMO and LUMO orbitals enhances ISC, promoting ROS generation. NCP effectively labels neutrophils and eradicates multidrug-resistant bacterial infections in mice (Figure 8D).

### 5.2. Stimulus-Responsive PSs for Spatiotemporally Precise Photodynamic Therapy

So far, a large number of highly efficient PSs have been developed that are not intelligent enough and lack the selectivity and specificity required to meet personalized treatment needs for tumor targeting. And PSs are always in an open state in the body, which has certain toxic side effects on healthy cells. The design strategy of activatable PSs is to connect quenchers to PSs through responsive chemical bonds; upon contact with the corresponding stimulus source, the quenchers that restrict PS fluorescence and photosensitization are specifically removed, allowing PSs to regain their emission and ROS production capabilities. Image-guided PDT can maximize the therapeutic efficiency of tumor areas while minimizing the side effects on healthy cells, which is necessary for enhancing PDT [112,113,114,115].

**Figure 9:**
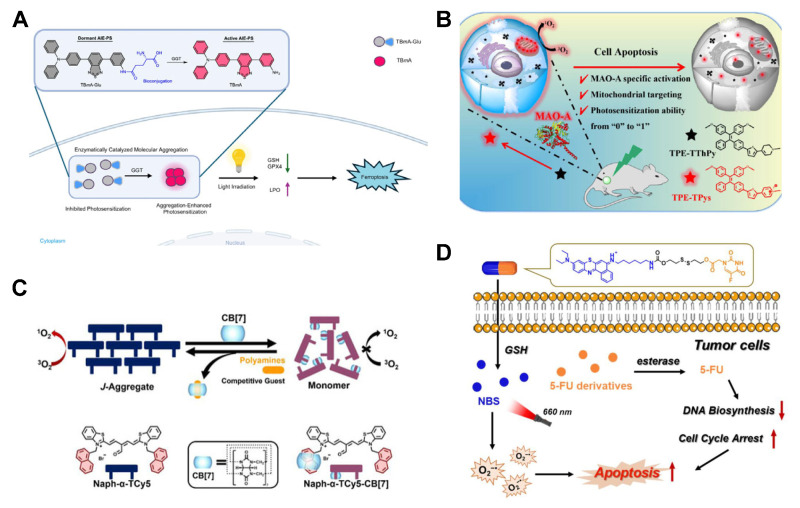
(**A**) Schematic diagram of the mechanism of GGT-activated AIE PS and their application in PDT. (**B**) Schematic diagram of monoamine oxidase A-activated AIE PS for tumor PDT. (**C**) Schematic diagram of ROS release by polyamine-activated supramolecular PS in cancer cells. (**D**) Schematic diagram of the PDT process GSH-activated PS in cancer cells.

Tan et al. [116] reported an AIE PS (TBMA) targeting gamma glutamyltransferase (GGT). GGT is an overexpressed enzyme in tumors that can specifically recognize and cleave the gamma glutamyltransferase bond in TBMA-Glu. The released TBMA, due to its insolubility in water, can emit bright fluorescence and restore photosensitivity in tumor aggregation. TBMA-Glu not only induces depletion of glutathione (GSH) through GGT photodegradation, but also induces lipid peroxidation accumulation and ferroptosis in tumor cells through photodynamic therapy (Figure 9A). Li et al. [117] developed an AIE PS (TPE-TThPy) activated by monoamine oxidase A (MAO-A). The MAO-A-mediated conversion of tetrahydropyridine (ThPy) to pyridinium salts (Pys) generates TPE-TTPys, which exhibits a D-π-A structure that narrows the HOMO-LUMO gap, promotes ISC, and enables red-shifted absorption/emission. This design allows precise imaging and treatment of MAO-A-overexpressing tumors in vivo and in vitro (Figure 9B).

In addition to biological enzymes, intracellular signaling molecules and peptides can also act as activators for PSs. Zhang et al. [118] reported a supramolecular PS (Naph-α-TCy5) activated by tumor-associated polyamines. In its monomeric state, Naph-α-TCy5 forms a complex with cucurbituril [7] urea (CB [7]), which suppresses ROS generation. However, when polyamines competitively bind to CB [7] in cancer cells, they disrupt the *J*-aggregate state of Naph-α-TCy5, enabling efficient ^1^O_2_ production. This system demonstrates potent antitumor activity and excellent biocompatibility (Figure 9C). Peng et al. [119] reported a NBS-2S-5FU specifically activated by the tumor microenvironment. Under the influence of GSH, the disulfide bond in NBS-2S-5FU is cleaved, leading to the release of NBS and chemotherapy agent 5-FU derivatives. Under irradiation, NBS generates sufficient O_2_^•−^, while 5-FU derivatives inhibit DNA biosynthesis, effectively suppressing tumor growth at low doses (Figure 9D).

This category of PSs activates their ROS-generating function only upon specific stimuli in the tumor microenvironment, which can theoretically greatly enhance the therapeutic precision. However, they are prone to issues such as false activation induced by single-stimulus triggers and heterogeneous distribution in tumor tissues, which remain key hurdles that must be overcome before their successful clinical translation.

### 5.3. Serum Protein-Enhanced PSs

Serum proteins, abundant in the body and non-toxic, serve as excellent drug carriers. They can enhance drug permeability and retention while exhibiting passive tumor-targeting properties. Composed of amino acids, serum proteins act as potential electron donors, functioning as electron pumps for PSs to promote their processes. Therefore, utilizing serum proteins in PDT can improve PS stability and safety, enable targeted drug delivery and imaging tracer transport, enhance therapeutic efficacy, and contribute to constructing efficient theranostic systems [120,121,122].

**Figure 10:**
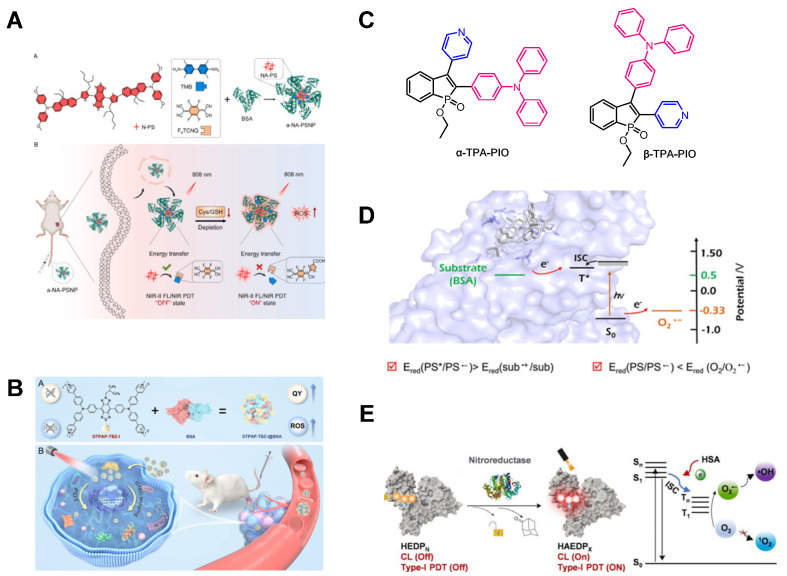
(**A**) Schematic diagram of a PS for PDT with activatable NIR-II luminescence using BSA as a nanocarrier. Reprinted with permission from Ref. [123]. Copyright 2024 Elsevier. (**B**) Schematic diagram of an AIE-active PS utilizing BSA as a nanocarrier for ultrahigh-resolution self-reporting PDT. Reprinted with permission from Ref. [124]. Copyright 2024 Elsevier. (**C**) Structural diagrams of two phosphine oxide-based type I PSs. (**D**) Schematic diagram of BSA-encapsulated PS enhancing type I PDT efficacy. Reprinted with permission from Ref. [126]. Copyright 2023 American Chemical Society. (**E**) Schematic diagram of PDT mediated by PS-HSA specific binding. Reprinted with permission from Ref. [127]. Copyright 2024 Wiley-VCH.

Liu et al. [123] reported a PS with activatable NIR-II luminescence using bovine serum albumin (BSA) as a nanocarrier. The PS (N-PS) binds to the charge transfer complex (CTC) in BSA, forming α-NA-PSNPs. Within BSA, aggregated N-PS exhibits high ROS yield and photostability. The CTC specifically responds to and consumes Cys/GSH (cysteine/glutathione), regulates the intracellular reducing environment, and promotes PDT. Upon CTC decomposition, the NIR-II fluorescence and ROS generation capability of α-NA-PS nanoparticles are restored under 808 nm laser irradiation, enabling fluorescence-guided PDT (Figure 10A). Tan et al. [124] developed a water-soluble PS (DTPAP-TBZ-I) with AIE characteristics in the short-wave infrared (SWIR) region, utilizing BSA as a nanocarrier for ultrahigh-resolution self-reporting PDT. Upon BSA encapsulation, DTPAP-TBZ-I exhibited a photoluminescence quantum yield of approximately 20.6% and an 18-fold enhancement in ROS generation. Serum proteins not only act as nanocarriers but also function as electron pumps for PSs, thereby enhancing their photosensitivity (Figure 10B). Tan et al. [125] reported two phosphine oxide-based type I PSs whose PDT efficacy was enhanced through BSA incorporation. The heavy atom effect of phosphine oxide promotes the ISC process and stabilizes external electrons to form free radical anion intermediates. By acting as an additional electron donor, BSA facilitates this mechanism, enabling type I ROS generation (Figure 10C). Peng et al. [126] demonstrated that BSA-encapsulated TADF PSs significantly enhance O_2_^•−^ production during type I PDT. TADF materials exhibit minimal Δ*E_ST_* values, allowing efficient ISC processes. BSA functions as an “electron reservoir” to promote free radical ion generation. The type I pathway demonstrates potent cytotoxicity against tumor cells under hypoxic conditions. Furthermore, by leveraging BSA’s tumor-targeting capability, PS@BSA achieved efficient PDT in tumor-bearing mice during in vivo experiments (Figure 10D). Pu et al. [127] designed near-infrared luminescent groups that specifically bind to human serum albumin (HSA), forming chemiluminescent protein complexes for PDT. Upon PS-HSA conjugation, HSA acts as an electron-rich carrier that participates in electron transfer reactions, suppresses non-radiative decay, amplifies chemiluminescence (CL) signals, and converts type II PDT to type I PDT. The HEDPO activates bright CL signals and PDT efficacy through nitroreductase (NTR)-catalyzed cleavage, a mechanism exploitable in hypoxic solid tumors with NTR overexpression (Figure 10E). Although serum protein carriers possess numerous advantages, their drawbacks are equally prominent, which are mainly manifested in the insufficient stability of the carriers themselves and limitations in delivery capacity, among other aspects. To address these issues, strategies such as constructing composite nanoparticles to improve stability and modifying serum proteins to enhance their targeting ability can be adopted.

### 5.4. Engineering Nano-PSs to Enhance PDT Efficacy

Among diverse phototherapeutic agents, nanoparticles have garnered significant attention for their on-demand activation, in situ functionality, and enhanced permeability and retention effects inherent to nanomaterials. Furthermore, organic fluorescent-group-doped nanoparticles offer structural flexibility and excellent biocompatibility. They can further integrate multiple functionalities, such as targeting capability, selective aggregation, and synergistic therapy, into a single system, thereby significantly enhancing therapeutic efficacy [128,129,130,131].

**Figure 11:**
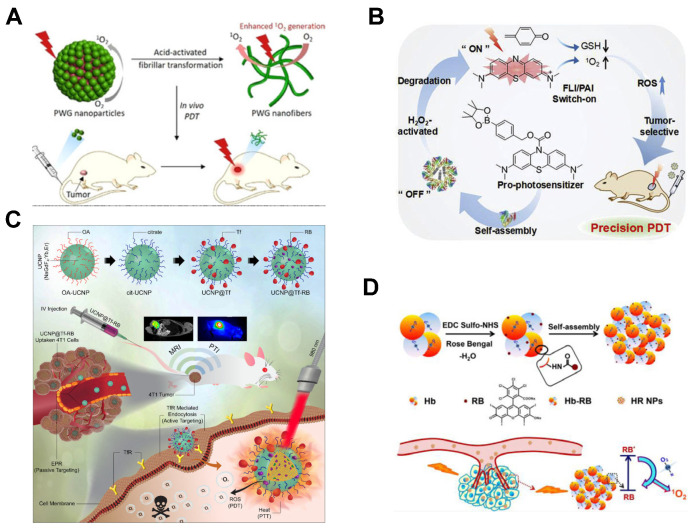
(**A**) Schematic diagram of pH responsive peptide nanoparticles for PDT of tumors. (**B**) H_2_O_2_ can activate nano PSs for PDT schematic diagram. Reprinted with permission from Ref. [133]. Copyright 2019 Elsevier. (**C**) Schematic diagram of synergistic photothermal and PDT of human transferrin (Tf) anchored on nanoparticles loaded with RB. Reprinted with permission from Ref. [134]. Copyright 2022 Elsevier. (**D**) Enhancement of ^1^O_2_ production by self-assembled PS nanoparticles of rose red and hemoglobin complexes. Reprinted with permission from Ref. [135]. Copyright 2022 Wiley-VCH.

Hest et al. [132] developed pH-responsive peptide-based nanoparticles. A tryptophan-glycine (WG) dipeptide was acylated to a hydrophobic porphyrin (P) core, forming a porphyrin-dipeptide conjugate (PWG). Glycine’s free carboxyl group enables acid-sensitive self-assembly into nanoparticles under physiological conditions. Upon entering tumor cells, environmental acidification triggers nanoparticle fibrillation. These nanofibers exhibit prolonged tumor retention and enhanced ^1^O_2_ generation, significantly improving PDT efficacy (Figure 11A). Xing et al. [133] designed H_2_O_2_-activatable biodegradable nanodrugs serving as PS prodrugs. The prodrug PS was encapsulated with BSA to formulate nanomedicine, which improves tumor-targeting selectivity, delivery efficiency, and cellular accumulation. H_2_O_2_-triggered decomposition activates the prodrug to release methylene blue (MB), restoring fluorescence/photoacoustic signals and photosensitizing activity for dual-modality imaging and ^1^O_2_ generation (Figure 11B). Chattopadhyay et al. [134] engineered tumor-targeted multifunctional nanoparticles by anchoring human transferrin (Tf) to NaGdF_4_:Yb, Er upconversion nanoparticles (UCNPs) loaded with rose bengal (RB). NIR excitation induces Förster resonance energy transfer (FRET) between UCNP and RB, generating ROS for PDT. Concurrently, non-radiative transitions in Er^3+^ produce localized heat for PTT. This synergistic PDT/PTT system enables rapid tumor suppression under multimodal imaging guidance (Figure 11C). Jie et al. [135] constructed hemoglobin (Hb)-RB complexes that self-assemble into highly photosensitive PS nanoparticles. Hb conjugates with RB via amide condensation and subsequently self-assembles into nanoparticles. The Hb-RB nanoparticles preserve Hb’s oxygen-binding capacity and undergo H_2_O_2_-triggered decomposition, elevating intratumoral oxygen levels while enhancing tumor accumulation. Even under ultralow-power-density light irradiation, these nanoparticles sustain robust ^1^O_2_ production (Figure 11D). At this stage, although nanoparticles are still hampered by issues such as insufficient practical delivery efficiency, constraints imposed by inherent material properties, and considerable difficulties in functional integration, the nanoparticle platform offers unprecedented capacity for functional integration, which makes it a pivotal pathway to drive the development of next-generation intelligent, activatable, and combination therapeutic phototheranostic agents.

## 6. Conclusions

Over the past decades, PDT has continuously advanced and evolved, with a number of PDT agents approved for clinical trials or clinical applications. However, PDT remains a non-mainstream option for cancer treatment, partly because the comprehensive performance of PSs has not yet met the standards for optimal therapeutic efficacy, leaving substantial room for improvement.

This paper systematically summarizes the key strategies for enhancing the efficacy of tumor PDT, focusing on three core directions: molecular structure modification of PSs, regulation of controllable aggregation behavior, and functionalized nanomaterial-mediated delivery. These strategies can effectively optimize the physicochemical and biological properties of PSs, enhance their targeted delivery efficiency and therapeutic controllability, and thereby significantly improve tumor treatment outcomes.

Despite the remarkable effectiveness of these strategies in improving PS performance, the clinical translation of PDT for cancer treatment still faces several major challenges: (1) PSs exhibit poor in vivo stability and are prone to inactivation or nonspecific aggregation in the complex biological microenvironment, leading to reduced therapeutic efficiency. To address this issue, novel materials such as AIE molecules and metal–organic frameworks can be developed as stable and efficient carrier systems to improve the in vivo behavior of PSs. (2) Severe hypoxia in deep tumor tissues greatly limits the efficacy of traditional PSs that rely on oxygen to exert their effects. The development of type I PSs can effectively reduce the oxygen dependence of therapy and break through the therapeutic bottleneck of hypoxic microenvironments. (3) The single therapeutic mode makes it difficult to achieve complete tumor elimination. Synergistic therapeutic systems can be constructed by combining PTT and photoacoustic therapy, or designing PSs with immune-regulatory functions, so as to improve the efficiency of tumor clearance. (4) Traditional PSs suffer from the drawback of single functionality and cannot simultaneously meet multiple requirements including water solubility, targeting ability, selective aggregation, and synergistic therapy. Through functional module design and nanotechnology, the multifunctional integration of PSs can be realized, which can specifically overcome the limitations of traditional PSs, endow them with multi-modal synergistic therapeutic capabilities, and promote a qualitative leap in therapeutic efficacy.

In summary, this work systematically reviews the core optimization strategies for PSs, aiming to provide clear design insights for enhancing the therapeutic efficacy of PDT. Meanwhile, it concisely summarizes the key challenges faced by PDT at the current stage and their potential solutions. We believe that with the collaborative efforts of researchers and clinicians, PDT is expected to evolve into one of the core modalities for cancer therapy in the future.

## Data Availability

No new data were created or analyzed in this study. Data sharing is not applicable to this article.

## Abbreviations

The following abbreviations are used in this manuscript:

ACQ	Aggregation-caused quenching
AIE	Aggregation-induced emission
ALP	Alkaline phosphatase
ASGPR	Asialoglycoprotein receptor
BODIPY	Boron-dipyrromethene
BSA	Serum albumin
CL	Chemiluminescence
CPT	Camptothecin
CTC	Charge transfer complex
Cys	Cysteine
FRET	Förster resonance energy transfer
GGT	Gamma glutamyltransferase
GSH	Glutathione
HAS	Human serum albumin
Hb	Hemoglobin
HOMO	Highest occupied molecular orbital
ICT	Intramolecular charge transfer
ISC	Intersystem crossing
LUMO	Lowest unoccupied molecular orbital
MB	Methylene blue
NIR	Near-infrared
NTR	Nitroreductase
OLEDs	Organic light-emitting diodes
PARP	Polymerase
PDT	Photodynamic therapy
PSs	Photosensitizers
PTT	Photothermal therapy
RB	Rose Bengal
RISC	Reverse intersystem crossing
RIM	Restricted intramolecular motion
ROS	Reactive oxygen species
SOC	Spin–orbit coupling
SWIR	Short-wave infrared
TADF	Thermally activated delayed fluorescence
TCF	Tricyanofuran
Tf	Transferrin

## References

[1] Siegel R.L., Miller K.D., Jemal A. (2020). Cancer statistics, 2020. CA Cancer J. Clin..

[2] Siegel R.L., Miller K.D., Wagle N.S., Jemal A. (2023). Cancer statistics, 2023. CA Cancer J. Clin..

[3] Xu D., Ge J., An Y., Bai S., Wang Z., Wu S., Dai Q., Lu Z., Liu G. (2023). Molecular Engineering of NIR-II/IIb Emitting AIEgen for Multimodal Imaging-Guided Photo-Immunotherapy. Small.

[4] Hu D., Pan M., Yu Y., Sun A., Shi K., Qu Y., Qian Z. (2020). Application of nanotechnology for enhancing photodynamic therapy via ameliorating, neglecting, or exploiting tumor hypoxia. VIEW.

[5] Li X., Lovell J.F., Yoon J., Chen X. (2020). Clinical development and potential of photothermal and photodynamic therapies for cancer. Nat. Rev. Clin. Oncol..

[6] Fan W., Yung B., Huang P., Chen X. (2017). Nanotechnology for Multimodal Synergistic Cancer Therapy. Chem. Rev..

[7] Li X., Lee S., Yoon J. (2018). Supramolecular photosensitizers rejuvenate photodynamic therapy. Chem. Soc. Rev..

[8] Li X., Kwon N., Guo T., Liu Z., Yoon J. (2018). Innovative Strategies for Hypoxic-Tumor Photodynamic Therapy. Angew. Chem. Int. Ed..

[9] Zhao J., Xu X., Yang Y., Li J. (2023). Assembled Photosensitizers Applied for Enhanced Photodynamic Therapy. CCS Chem..

[10] Meng Z., Zhou X., Xu J., Han X., Dong Z., Wang H., Zhang Y., She J., Xu L., Wang C. (2019). Light-Triggered In Situ Gelation to Enable Robust Photodynamic-Immunotherapy by Repeated Stimulations. Adv. Mater..

[11] Liu J., Liu T., Du P., Zhang L., Lei J. (2019). Metal–Organic Framework (MOF) Hybrid as a Tandem Catalyst for Enhanced Therapy against Hypoxic Tumor Cells. Angew. Chem. Int. Ed..

[12] Solban N., Rizvi I., Hasan T. (2006). Targeted photodynamic therapy. Lasers Surg. Med..

[13] Filatov M.A. (2020). Heavy-atom-free BODIPY photosensitizers with intersystem crossing mediated by intramolecular photoinduced electron transfer. Org. Biomol. Chem..

[14] Li J., Zhuang Z., Zhao Z., Tang B.Z. (2022). Type I AIE photosensitizers: Mechanism and application. VIEW.

[15] Amos-Tautua B.M., Songca S.P., Oluwafemi O.S. (2019). Application of Porphyrins in Antibacterial Photodynamic Therapy. Molecules.

[16] Ma Y., Liu X., Jiang Q., Xu H.-D., Liang G., Zhan W., Sun X., Liang G. (2025). Cell Membrane-Anchored Click Reaction Enhances Porphyrin Uptake for Highly Efficient Photodynamic Therapy of Breast Tumors. J. Am. Chem. Soc..

[17] Liu B.-K., Teng K.-X., Niu L.-Y., Yang Q.-Z. (2025). BODIPY-Based Photosensitizer with Outstanding Photocytotoxicity for Deep-Tissue Photodynamic Therapy. ACS Mater. Lett..

[18] Bureller L., Michelin C., Cisnetti F. (2025). Synthesis of BODIPY@MOFs as Hybrid Materials for Emerging Applications: A Review. Molecules.

[19] He L., Ma D. (2024). Self-assembled phthalocyanine-based nano-photosensitizers in photodynamic therapy for hypoxic tumors. Mater. Chem. Front..

[20] Chornovolenko K., Koczorowski T. (2025). Phthalocyanines Conjugated with Small Biologically Active Compounds for the Advanced Photodynamic Therapy: A Review. Molecules.

[21] Nash G.T., Luo T., Lan G., Ni K., Kaufmann M., Lin W. (2021). Nanoscale Metal–Organic Layer Isolates Phthalocyanines for Efficient Mitochondria-Targeted Photodynamic Therapy. J. Am. Chem. Soc..

[22] Tavakkoli Yaraki M., Liu B., Tan Y.N. (2022). Emerging Strategies in Enhancing Singlet Oxygen Generation of Nano-Photosensitizers Toward Advanced Phototherapy. Nano-Micro Lett..

[23] Yang G., Lu S.-B., Li C., Chen F., Ni J.-S., Zha M., Li Y., Gao J., Kang T., Liu C. (2021). Type I macrophage activator photosensitizer against hypoxic tumors. Chem. Sci..

[24] Ma N., Wang J., Tang H., Wu S., Liu X., Chen K., Zhang Y., Yu X. (2025). The Current Advances in Design Strategy (Indirect Strategy and Direct Strategy) for Type-I Photosensitizers. Adv. Sci..

[25] Han H.-H., Wang H.-M., Jangili P., Li M., Wu L., Zang Y., Sedgwick A.C., Li J., He X.-P., James T.D. (2023). The design of small-molecule prodrugs and activatable phototherapeutics for cancer therapy. Chem. Soc. Rev..

[26] Teng Y., Wang D., Yang Z., Wang R., Ning S., Zhang R., Yang H., Feng X., Liu J., Yang L. (2025). Bioorthogonal strategy-triggered In situ co-activation of aggregation-induced emission photosensitizers and chemotherapeutic prodrugs for boosting synergistic chemo-photodynamic-immunotherapy. Biomaterials.

[27] Hu J., Wu D., Pan Q., Li H., Zhang J., Geng F. (2022). Recent Development of Photoresponsive Liposomes Based on Organic Photosensitizers, Au Nanoparticles, and Azobenzene Derivatives for Nanomedicine. ACS Appl. Nano Mater..

[28] Huo M., Liu P., Zhang L., Wei C., Wang L., Chen Y., Shi J. (2021). Upconversion Nanoparticles Hybridized Cyanobacterial Cells for Near-Infrared Mediated Photosynthesis and Enhanced Photodynamic Therapy. Adv. Funct. Mater..

[29] Lima E., Reis L.V. (2023). Photodynamic Therapy: From the Basics to the Current Progress of N-Heterocyclic-Bearing Dyes as Effective Photosensitizers. Molecules.

[30] Zhao Y., Duan R., Zhao J., Li C. (2018). Spin–orbit charge transfer intersystem crossing in perylenemonoimide–phenothiazine compact electron donor–acceptor dyads. Chem. Commun..

[31] Sasikumar D., John A.T., Sunny J., Hariharan M. (2020). Access to the triplet excited states of organic chromophores. Chem. Soc. Rev..

[32] Wu W., Shi L., Duan Y., Xu S., Shen L., Zhu T., Hou L., Meng X., Liu B. (2021). Nanobody modified high-performance AIE photosensitizer nanoparticles for precise photodynamic oral cancer therapy of patient-derived tumor xenograft. Biomaterials.

[33] Ni J., Wang Y., Zhang H., Sun J.Z., Tang B.Z. (2021). Aggregation-Induced Generation of Reactive Oxygen Species: Mechanism and Photosensitizer Construction. Molecules.

[34] Yuan R., Chen W., Zhuang M., Chi X., Ma L., Mi L., Dong M., Huang P., Wan Y., Zhang P. (2025). Tröger’s Base as a Potential Bridge to Type-I Photosensitizers: Mechanism and Antitumor Applications. J. Med. Chem..

[35] Fan X., Lv S., Lv F., Feng E., Liu D., Zhou P., Song F. (2024). Type-I Photodynamic Therapy Induced by Pt-Coordination of Type-II Photosensitizers into Supramolecular Complexes. Chem. Eur. J..

[36] Wen H., Wu Q., Xiang X., Sun T., Xie Z., Chen X. (2024). PEGylated BODIPY Photosensitizer for Type I Dominant Photodynamic Therapy and Afterglow Imaging. ACS Appl. Mater. Interfaces.

[37] Shi M., Pan W., Gao P., Chen Y., Wang K., Li N., Tang B. (2023). A protein-targeted photosensitizer for highly efficient cancer therapy. Mater. Today Chem..

[38] Tian Y., Wang L., Qian Y., Li L., Li H., Song X., Wang M., Zhu N., Tong Y., Wu W. (2024). DNA-Targeting Bioactive Photosensitizer for Chemo-Photodynamic Therapy of Malignant Tumor. ACS Mater. Lett..

[39] Qi Y., Wang H., Du A., Liu C., Sun X., Meng X., Shen J., Zhang S.-Y., Zhang L.-R., Jiang B. (2023). Engineering Multifunctional Thylakoid as an Oxygen Self-Supplying Photosensitizer for Esophageal Squamous Cell Carcinoma-Targeted Photodynamic Therapy. CCS Chem..

[40] Qu R., Zhen X., Jiang X. (2021). Emerging Designs of Aggregation-Induced Emission Agents for Enhanced Phototherapy Applications. CCS Chem..

[41] Guo X., Yang N., Ji W., Zhang H., Dong X., Zhou Z., Li L., Shen H.-M., Yao S.Q., Huang W. (2021). Mito-Bomb: Targeting Mitochondria for Cancer Therapy. Adv. Mater..

[42] Qin L., Zhao C., Yao L.-Y., Dou H., Zhang M., Xie J., Weng T.-C., Lv H., Yang G.-Y. (2021). Efficient Photogeneration of Hydrogen Boosted by Long-Lived Dye-Modified Ir(III) Photosensitizers and Polyoxometalate Catalyst. CCS Chem..

[43] Bartusik-Aebisher D., Przygórzewska A., Woźnicki P., Aebisher D. (2025). Nanoparticles for Photodynamic Therapy of Breast Cancer: A Review of Recent Studies. Molecules.

[44] Michalak M., Szymczyk J., Pawska A., Wysocki M., Janiak D., Ziental D., Ptaszek M., Güzel E., Sobotta L. (2025). Chlorin Activity Enhancers for Photodynamic Therapy. Molecules.

[45] Pham T.C., Nguyen V.-N., Choi Y., Lee S., Yoon J. (2021). Recent Strategies to Develop Innovative Photosensitizers for Enhanced Photodynamic Therapy. Chem. Rev..

[46] He M., Xiao M., Wang R., Fan J., Peng X., Sun W. (2025). Phototherapeutic nanoagents for cancer immunotherapy. Prog. Mater. Sci..

[47] Gao J., Tian Y., Li Y., Hu F., Wu W. (2024). Design strategies for aggregation-induced emission photosensitizers with enhanced safety in photodynamic therapy. Coord. Chem. Rev..

[48] Wang R., Hua S., Xing Y., Wang R., Wang H., Jiang T., Yu F. (2024). Organic dye-based photosensitizers for fluorescence imaging-guided cancer phototheranostics. Coord. Chem. Rev..

[49] Surur A.K., de Oliveira A.B., De Annunzio S.R., Ferrisse T.M., Fontana C.R. (2024). Bacterial resistance to antimicrobial photodynamic therapy: A critical update. J. Photochem. Photobiol. B Biol..

[50] Wu W., Liu B. (2022). Modulating the optical properties and functions of organic molecules through polymerization. Mater. Horiz..

[51] Akbar A., Khan S., Chatterjee T., Ghosh M. (2023). Unleashing the power of porphyrin photosensitizers: Illuminating breakthroughs in photodynamic therapy. J. Photochem. Photobiol. B Biol..

[52] Xu S., Yuan Y., Cai X., Zhang C.-J., Hu F., Liang J., Zhang G., Zhang D., Liu B. (2015). Tuning the singlet-triplet energy gap: A unique approach to efficient photosensitizers with aggregation-induced emission (AIE) characteristics. Chem. Sci..

[53] Chou P.-T., Chi Y., Chung M.-W., Lin C.-C. (2011). Harvesting luminescence via harnessing the photophysical properties of transition metal complexes. Coord. Chem. Rev..

[54] Pang E., Zhao S., Wang B., Niu G., Song X., Lan M. (2022). Strategies to construct efficient singlet oxygen-generating photosensitizers. Coord. Chem. Rev..

[55] Kamkaew A., Lim S.H., Lee H.B., Kiew L.V., Chung L.Y., Burgess K. (2013). BODIPY dyes in photodynamic therapy. Chem. Soc. Rev..

[56] Ouyang J., Li D., Zhu L., Cai X., Liu L., Pan H., Ma A. (2024). Application and Challenge of Metalloporphyrin Sensitizers in Noninvasive Dynamic Tumor Therapy. Molecules.

[57] Karges J., Basu U., Blacque O., Chao H., Gasser G. (2019). Polymeric Encapsulation of Novel Homoleptic Bis(dipyrrinato) Zinc(II) Complexes with Long Lifetimes for Applications as Photodynamic Therapy Photosensitisers. Angew. Chem. Int. Ed..

[58] Yue Y., Li B., Wang D., Wu C., Li Z., Liu B. (2024). Optimizing Photosensitizers with Type I and Type II ROS Generation Through Modulating Triplet Lifetime and Intersystem Crossing Efficiency. Adv. Funct. Mater..

[59] Zhang Y., Zhao M., Miao J., Gu W., Zhu J., Cheng B., Li Q., Miao Q. (2023). Hemicyanine-Based Type I Photosensitizers for Antihypoxic Activatable Photodynamic Therapy. ACS Mater. Lett..

[60] Xiao X., Zhao X., Chen X., Zhao J. (2023). Heavy Atom-Free Triplet Photosensitizers: Molecular Structure Design, Photophysical Properties and Application in Photodynamic Therapy. Molecules.

[61] Chen K.-K., Guo S., Ding M.-J., Lu T.-B., Zhang Z.-M. (2023). Heavy-Atom-Free Photosensitizers for High-Yield CO_2_-to-CO Conversion. CCS Chem..

[62] Zhang F., Cai H., Wang L., Shao J. (2024). Synthesis of heavy-atom-free thienoisoindigo dye as near-infrared photosensitizer for type I photodynamic therapy and photoacoustic imaging. J. Photochem. Photobiol. B Biol..

[63] Tang J., Wang L., Loredo A., Cole C., Xiao H. (2020). Single-atom replacement as a general approach towards visible-light/near-infrared heavy-atom-free photosensitizers for photodynamic therapy. Chem. Sci..

[64] Liu W., He S., Ma X., Lv C., Gu H., Cao J., Du J., Sun W., Fan J., Peng X. (2024). Near–Infrared Heptamethine Cyanine Photosensitizers with Efficient Singlet Oxygen Generation for Anticancer Photodynamic Therapy. Angew. Chem. Int. Ed..

[65] Sasaki Y., Yanai N., Kimizuka N. (2022). Osmium Complex–Chromophore Conjugates with Both Singlet-to-Triplet Absorption and Long Triplet Lifetime through Tuning of the Heavy-Atom Effect. Inorg. Chem..

[66] Xu G., Li C., Chi C., Wu L., Sun Y., Zhao J., Xia X.-H., Gou S. (2022). A supramolecular photosensitizer derived from an Arene-Ru(II) complex self-assembly for NIR activated photodynamic and photothermal therapy. Nat. Commun..

[67] Zhang P., Chiu C.K.C., Huang H., Lam Y.P.Y., Habtemariam A., Malcomson T., Paterson M.J., Clarkson G.J., O’Connor P.B., Chao H. (2017). Organoiridium Photosensitizers Induce Specific Oxidative Attack on Proteins within Cancer Cells. Angew. Chem. Int. Ed..

[68] Nguyen V.-N., Yim Y., Kim S., Ryu B., Swamy K.M.K., Kim G., Kwon N., Kim C.Y., Park S., Yoon J. (2020). Molecular Design of Highly Efficient Heavy-Atom-Free Triplet BODIPY Derivatives for Photodynamic Therapy and Bioimaging. Angew. Chem. Int. Ed..

[69] Zheng Z., Liu H., Zhai S., Zhang H., Shan G., Kwok R.T.K., Ma C., Sung H.H.Y., Williams I.D., Lam J.W.Y. (2020). Highly efficient singlet oxygen generation, two-photon photodynamic therapy and melanoma ablation by rationally designed mitochondria-specific near-infrared AIEgens. Chem. Sci..

[70] Zhao X., Yao Q., Long S., Chi W., Yang Y., Tan D., Liu X., Huang H., Sun W., Du J. (2021). An Approach to Developing Cyanines with Simultaneous Intersystem Crossing Enhancement and Excited-State Lifetime Elongation for Photodynamic Antitumor Metastasis. J. Am. Chem. Soc..

[71] Hao Y., Wang J., Wang J., Guo Z., Liu X., Lv S., Hu Q., Huo K., Yao Q., Jiang J. (2024). Leveraging an Electron-Acceptor Engineering Strategy to Regulate Excitation Dynamics of Dyes for Devising Ideal Phototherapeutic Agents in Synergistic Photodynamic/Mild-Photothermal Tumor Therapy. CCS Chem..

[72] Huang H., Liu Q., Zhu J.-H., Tong Y., Huang D., Zhou D., Long S., Wang L., Li M., Chen X. (2024). Dimerized Pentamethine Cyanines for Synergistically Boosting Photodynamic/Photothermal Therapy. Aggregate.

[73] Fang F., Zhu L., Li M., Song Y., Sun M., Zhao D., Zhang J. (2021). Thermally Activated Delayed Fluorescence Material: An Emerging Class of Metal-Free Luminophores for Biomedical Applications. Adv. Sci..

[74] Zhu Z., Zhang X., Guo X., Wu Q., Li Z., Yu C., Hao E., Jiao L., Zhao J. (2021). Orthogonally aligned cyclic BODIPY arrays with long-lived triplet excited states as efficient heavy-atom-free photosensitizers. Chem. Sci..

[75] Chen J.-X., Tao W.-W., Xiao Y.-F., Tian S., Chen W.-C., Wang K., Yu J., Geng F.-X., Zhang X.-H., Lee C.-S. (2019). Isomeric thermally activated delayed fluorescence emitters based on indolo[2,3-b]acridine electron-donor: A compromising optimization for efficient orange–red organic light-emitting diodes. J. Mater. Chem. C.

[76] Zhang J., Chen W., Chen R., Liu X.-K., Xiong Y., Kershaw S.V., Rogach A.L., Adachi C., Zhang X., Lee C.-S. (2016). Organic nanostructures of thermally activated delayed fluorescent emitters with enhanced intersystem crossing as novel metal-free photosensitizers. Chem. Commun..

[77] Zhang J., Fang F., Liu B., Tan J.-H., Chen W.-C., Zhu Z., Yuan Y., Wan Y., Cui X., Li S. (2019). Intrinsically Cancer-Mitochondria-Targeted Thermally Activated Delayed Fluorescence Nanoparticles for Two-Photon-Activated Fluorescence Imaging and Photodynamic Therapy. ACS Appl. Mater. Interfaces.

[78] Song J., Fang H., Wang X., Zhong W. (2024). TADF-Guiding Modification of Endoplasmic Reticulum-Targeted Photosensitizers for Efficient Photodynamic Immunotherapy. Small.

[79] Barman D., Rajamalli P., Bidkar A.P., Sarmah T., Ghosh S.S., Zysman-Colman E., Iyer P.K. (2025). Modulation of Donor in Purely Organic Triplet Harvesting AIE-TADF Photosensitizer for Image-guided Photodynamic Therapy. Small.

[80] Zang Q., Yu J., Yu W., Qian J., Hu R., Tang B.Z. (2018). Red-emissive azabenzanthrone derivatives for photodynamic therapy irradiated with ultralow light power density and two-photon imaging. Chem. Sci..

[81] Huang H., Ma D., Liu Q., Huang D., Zhao X., Yao Q., Xiong T., Long S., Du J., Fan J. (2021). Enhancing Intersystem Crossing by Intermolecular Dimer-Stacking of Cyanine as Photosensitizer for Cancer Therapy. CCS Chem..

[82] Chen C., Zhang R., Zhang J., Zhang Y., Zhang H., Wang Z., Huang X., Chen S., Kwok Ryan T.K., Lam Jacky W.Y. (2021). Taming Reactive Oxygen Species: Mitochondria-Targeting Aggregation-Induced Emission Luminogen for Neuron Protection via Photosensitization-Triggered Autophagy. CCS Chem..

[83] Gao Y., Chen X., Li M., Zhang L., Zhu J. (2022). Photonic Crystal-Enhanced Photodynamic Antibacterial Therapy. CCS Chem..

[84] Liu S., Zhang H., Li Y., Liu J., Du L., Chen M., Kwok R.T.K., Lam J.W.Y., Phillips D.L., Tang B.Z. (2018). Strategies to Enhance the Photosensitization: Polymerization and the Donor–Acceptor Even–Odd Effect. Angew. Chem. Int. Ed..

[85] Xiong W., Wang L., Chen X., Tang H., Cao D., Zhang G., Chen W. (2020). Pyridinium-substituted tetraphenylethylene salt-based photosensitizers by varying counter anions: A highly efficient photodynamic therapy for cancer cell ablation and bacterial inactivation. J. Mater. Chem. B.

[86] Su H., Shang W., Li G., Yan W., Yan X., Tang B.Z., Qin W. (2024). Near-Infrared II AIE Luminogens with Mitochondria-Targeting Characteristics for Combinational Phototherapies of Breast Tumors Through Synergistic Cell Apoptosis and Pyroptosis. Adv. Funct. Mater..

[87] Li Y., Ma T., Jiang H., Li W., Tian D., Zhu J., Li Z.a. (2022). Anionic Cyanine J-Type Aggregate Nanoparticles with Enhanced Photosensitization for Mitochondria-Targeting Tumor Phototherapy. Angew. Chem. Int. Ed..

[88] Sun Z., Wang J., Xiao M., Wu K., Wang C., Fu H., Lv S., Shi L., Zhu C. (2024). A straightforward strategy to modulate ROS generation of AIE photosensitizers for type-I PDT. Chem. Eng. J..

[89] Hu X., Zhang C., Lv J., Li R., Qin A., Zhu C., Sun F., Chen Z., Teng S., Lin H. (2025). Constructing Intramolecular Electric Fields in NIR-II-Emissive Photosensitizers to Regulate the Local Electron Density for Boosting Hypoxia-Tolerant Cancer Phototheranostics. CCS Chem..

[90] Li C., Li J., Pang Y., Mei L., Xu W., Zhang Z., Han C., Sun Y. (2024). Harnessing donor cyclization strategy: Converting type II to type I photosensitizers and enhancing AIE performance for NIR-II FL/MR imaging-guided photodynamic therapy under hypoxia condition. Chem. Eng. J..

[91] Sun T., Wang S., Liu X., Ji D., Xie X., Yang R., Wang L., Ling Y., Ling C.-C. (2025). Novel ꞵ-carboline/cyanoisoflavone photosensitizers for ferroptosis-induced efficient chemo-photodynamic synergistic cancer therapy. J. Photochem. Photobiol. B Biol..

[92] Liang J., Ran X., Liu Y., Yu X., Chen S., Li K. (2024). Rational design of type-I photosensitizer molecules for mitochondrion-targeted photodynamic therapy. J. Mater. Chem. B.

[93] Zhen S., Xu Z., Suo M., Zhang T., Lyu M., Li T., Zhang T., Li M., Zhao Z., Tang B.Z. (2024). NIR-II AIE Liposomes for Boosting Type-I Photodynamic and Mild-Temperature Photothermal Therapy in Breast Cancer Treatment. Adv. Mater..

[94] Xiang C., Liu Y., Ding Q., Jiang T., Li C., Xiang J., Yang X., Yang T., Wang Y., Tan Y. (2024). Precise Molecular Engineering of Multi-Suborganelle Targeted NIR Type-I AIE Photosensitizer and Design of Cell Membrane-Anchored Anti-Tumor Pyroptosis Vaccine. Adv. Funct. Mater..

[95] An J., Tang S., Hong G., Chen W., Chen M., Song J., Li Z., Peng X., Song F., Zheng W.-H. (2022). An unexpected strategy to alleviate hypoxia limitation of photodynamic therapy by biotinylation of photosensitizers. Nat. Commun..

[96] Zhuang J., Liu S., Li B., Li Z., Wu C., Xu D., Pan W., Li Z., Liu X., Liu B. (2024). Electron Transfer Mediator Modulates Type II Porphyrin-Based Metal–Organic Framework Photosensitizers for Type I Photodynamic Therapy. Angew. Chem. Int. Ed..

[97] Zhuang J., Qi G., Feng Y., Wu M., Zhang H., Wang D., Zhang X., Chong K.C., Li B., Liu S. (2024). Thymoquinone as an electron transfer mediator to convert Type II photosensitizers to Type I photosensitizers. Nat. Commun..

[98] Chang R., Nikoloudakis E., Zou Q., Mitraki A., Coutsolelos A.G., Yan X. (2020). Supramolecular Nanodrugs Constructed by Self-Assembly of Peptide Nucleic Acid–Photosensitizer Conjugates for Photodynamic Therapy. ACS Appl. Bio Mater..

[99] Chen X., Dong S., Chen J., Ye Q., Cao S., Wang K., Peng Y. (2025). Dual-color imaging of lipid droplets and lysosomes with triphenylamine-phenyl silicon phthalocyanine nanoprobes for enhanced photodynamic therapy. J. Photochem. Photobiol. B Biol..

[100] Cheng H.-B., Qiao B., Li H., Cao J., Luo Y., Kotraiah Swamy K.M., Zhao J., Wang Z., Lee J.Y., Liang X.-J. (2021). Protein-Activatable Diarylethene Monomer as a Smart Trigger of Noninvasive Control Over Reversible Generation of Singlet Oxygen: A Facile, Switchable, Theranostic Strategy for Photodynamic-Immunotherapy. J. Am. Chem. Soc..

[101] Wang R., Kim K.-H., Yoo J., Li X., Kwon N., Jeon Y.-H., Shin S.-K., Han S.S., Lee D.-S., Yoon J. (2022). A Nanostructured Phthalocyanine/Albumin Supramolecular Assembly for Fluorescence Turn-On Imaging and Photodynamic Immunotherapy. ACS Nano.

[102] Han J., Li H., Zhao L., Kim G., Chen Y., Yan X., Yoon J. (2022). Albumin-mediated “Unlocking” of supramolecular prodrug-like nanozymes toward selective imaging-guided phototherapy. Chem. Sci..

[103] Zhang Q., Liu N., Wang J., Liu Y., Wang K., Zhang J., Pan X. (2023). The Recent Advance of Cell-Penetrating and Tumor-Targeting Peptides as Drug Delivery Systems Based on Tumor Microenvironment. Mol. Pharm..

[104] Chen J., Li S., Wang Z., Pan Y., Wei J., Lu S., Zhang Q.-W., Wang L.-H., Wang R. (2021). Synthesis of an AIEgen functionalized cucurbit[7]uril for subcellular bioimaging and synergistic photodynamic therapy and supramolecular chemotherapy. Chem. Sci..

[105] Porubský M., Hodoň J., Stanková J., Džubák P., Hajdúch M., Urban M., Hlaváč J. (2024). Near-infrared pH-switchable BODIPY photosensitizers for dual biotin/cRGD targeted photodynamic therapy. J. Photochem. Photobiol. B Biol..

[106] Gierlich P., Mata A.I., Donohoe C., Brito R.M.M., Senge M.O., Gomes-da-Silva L.C. (2020). Ligand-Targeted Delivery of Photosensitizers for Cancer Treatment. Molecules.

[107] Tao J., Yuan Z., Zhou M. (2025). Targeted photodynamic therapy: Enhancing efficacy through specific organelle engagement. Front. Pharmacol..

[108] Fan L., Zhang P., Gan Y., Li L., Wang L., Wang M., Liu X., Ma J., Liu B., Shi L. (2024). A Molecular-Targeting Photosensitizer to Inhibit DNA Damage Repair System and Induce cGAS-STING Pathway Activation for Photo-Immunotherapy of Ovarian Cancer. Adv. Funct. Mater..

[109] Song J.W., Ahn J.W., Lee M.W., Kim H.J., Kang D.O., Kim R.H., Kang U.G., Kim Y.H., Han J., Park Y.H. (2021). Targeted theranostic photoactivation on atherosclerosis. J. Nanobiotechnol..

[110] Zhou W., Liu Y.-C., Liu G.-J., Zhang Y., Feng G.-L., Xing G.-W. (2024). Glycosylated AIE-active Red Light-triggered Photocage with Precisely Tumor Targeting Capability for Synergistic Type I Photodynamic Therapy and CPT Chemotherapy. Angew. Chem. Int. Ed..

[111] Wang P., Bai Q., Liu X., Zhao M., Chen L., Hu F., Ye J., Chen X., Wang K.-N., Liu B. (2024). Nucleus-Targeting Photosensitizers Enhance Neutrophil Extracellular Traps for Efficient Eradication of Multidrug-Resistant Bacterial Infections. Adv. Mater..

[112] Lang W., Chen L.-Z., Chen Y., Cao Q.-Y. (2023). A GSH-activated AIE-based polymer photosensitizer for killing cancer cells. Talanta.

[113] Min X., Yi F., Han X.-L., Li M., Gao Q., Liang X., Chen Z., Sun Y., Liu Y. (2022). Targeted photodynamic therapy using a water-soluble aggregation-Induced emission photosensitizer activated by an acidic tumor microenvironment. Chem. Eng. J..

[114] Yang X., Xu C., Zhang X., Li P., Sun F., Liu X., Wang X., Kwok R.T.K., Yang J., Lam J.W.Y. (2023). Development of Sulfonamide-Functionalized Charge-Reversal AIE Photosensitizers for Precise Photodynamic Therapy in the Acidic Tumor Microenvironment. Adv. Funct. Mater..

[115] Yu Q., Li J., Yu Y., Yan M., Xu D., Yin S. (2024). Biomarker-activatable photosensitizers with aggregation-induced emission characteristics for photodynamic therapy. Coord. Chem. Rev..

[116] Wang W.-J., Zhang R., Zhang L., Hao L., Cai X.-M., Wu Q., Qiu Z., Han R., Feng J., Wang S. (2024). Enzymatically catalyzed molecular aggregation. Nat. Commun..

[117] Hu Y., Yin S.-Y., Liu W., Li Z., Chen Y., Li J. (2023). Rationally designed monoamine oxidase A-activatable AIE molecular photosensitizer for the specific imaging and cellular therapy of tumors. Aggregate.

[118] Ma H., Xu W., Tang X., Kang Y., Xu J.-F., Zhang X. (2025). A Supramolecularly Activatable Photosensitizer: Controllable Cyanine J-Aggregation for Efficient Photodynamic Therapy. CCS Chem..

[119] Huang D., Yin J., Zou Y., Huang H., Long S., Sun W., Du J., Fan J., Peng X. (2024). A self-reinforced activatable photosensitizer prodrug enabling synergistic photodynamic and chemotherapy. Smart Mol..

[120] Li X., Li X., Park S., Wu S., Guo Y., Nam K.T., Kwon N., Yoon J., Hu Q. (2024). Photodynamic and Photothermal therapy via human serum albumin delivery. Coord. Chem. Rev..

[121] Mattioli E.J., Ulfo L., Marconi A., Pellicioni V., Costantini P.E., Marforio T.D., Di Giosia M., Danielli A., Fimognari C., Turrini E. (2023). Carrying Temoporfin with Human Serum Albumin: A New Perspective for Photodynamic Application in Head and Neck Cancer. Biomolecules.

[122] Wang W., Wang Y., Ma M., Jin H.J., Li X. (2022). Drug-Induced Self-Assembly Cascade Nanoreactor for Synergistic Tumor Therapy. ACS Appl. Mater. Interfaces.

[123] Wu K., Liu J., Zhang X., Chao Z., Fang Y., Zhu Y., Liu Y., Zhang X., Wang Q., Ju H. (2025). Bovine serum albumin framed activatable NIR AIE photosensitizer for targeted tumor therapy. Biomaterials.

[124] Xu Y., Zhang J., Wang Z., Zhang P., Zhang Z., Yang Z., Lam J.W.Y., Kwok R.T.K., Meng L., Dang D. (2025). Water-soluble AIE photosensitizer in short-wave infrared region for albumin-enhanced and self-reporting phototheranostics. Biomaterials.

[125] Zhuang Z., Dai J., Yu M., Li J., Shen P., Hu R., Lou X., Zhao Z., Tang B.Z. (2020). Type I photosensitizers based on phosphindole oxide for photodynamic therapy: Apoptosis and autophagy induced by endoplasmic reticulum stress. Chem. Sci..

[126] Chen W., Wang Z., Tian M., Hong G., Wu Y., Sui M., Chen M., An J., Song F., Peng X. (2023). Integration of TADF Photosensitizer as “Electron Pump” and BSA as “Electron Reservoir” for Boosting Type I Photodynamic Therapy. J. Am. Chem. Soc..

[127] Huang J., Liu J., Wu J., Xu M., Lin Y., Pu K. (2024). Near-Infrared Chemiluminophore Switches Photodynamic Processes via Protein Complexation for Biomarker-Activatable Cancer Therapy. Angew. Chem. Int. Ed..

[128] Li X., Wang J., Cui R., Xu D., Zhu L., Li Z., Chen H., Gao Y., Jia L. (2020). Hypoxia/pH dual-responsive nitroimidazole-modified chitosan/rose bengal derivative nanoparticles for enhanced photodynamic anticancer therapy. Dye. Pigment..

[129] Zeng Q., Li X., Xie S., Xing D., Zhang T. (2022). Specific disruption of glutathione-defense system with activatable single molecule-assembled nanoprodrug for boosted photodynamic/chemotherapy eradication of drug-resistant tumors. Biomaterials.

[130] Yang H., Liu R., Xu Y., Qian L., Dai Z. (2021). Photosensitizer Nanoparticles Boost Photodynamic Therapy for Pancreatic Cancer Treatment. Nano-Micro Lett..

[131] Wu J., Zong D., Li F. (2025). Enhanced photodynamic therapy with riboflavin@ dual minerals doped hydroxyapatite nanoparticles: A promising in vitro approach for bladder cancer. J. Photochem. Photobiol. B Biol..

[132] Sun B., Chang R., Cao S., Yuan C., Zhao L., Yang H., Li J., Yan X., van Hest J.C.M. (2020). Acid-Activatable Transmorphic Peptide-Based Nanomaterials for Photodynamic Therapy. Angew. Chem. Int. Ed..

[133] Zeng Q., Zhang R., Zhang T., Xing D. (2019). H_2_O_2_-responsive biodegradable nanomedicine for cancer-selective dual-modal imaging guided precise photodynamic therapy. Biomaterials.

[134] Akhtar N., Chen C.L., Chattopadhyay S. (2022). PDT-active upconversion nanoheaters for targeted imaging guided combinatorial cancer phototherapies with low-power single NIR excitation. Biomater. Adv..

[135] Zhao J., Yang Y., Xu X., Li H., Fei J., Liu Y., Zhang X., Li J. (2022). Super Light-Sensitive Photosensitizer Nanoparticles for Improved Photodynamic Therapy against Solid Tumors. Angew. Chem. Int. Ed..

